# Early Orthodontic and Orthopedic Interventions for Anterior Open Bite in the Mixed Dentition: A Systematic Review

**DOI:** 10.7759/cureus.96806

**Published:** 2025-11-13

**Authors:** Asma Salih Fadul Yousif, Ahmed Fathi Farah Hassan, Leina Tarig Mohamed Ali Idress, Israa K B Elhassan, Rania Elnour, Eman Ali Mohamed Salih Omer

**Affiliations:** 1 Department of Orthodontics, University of Khartoum, Khartoum, SDN; 2 Department of Dentistry, Najran Armed Forces Hospital, Ministry of Defense Health Services, Najran, SAU; 3 Department of Orthodontics, Ajman University, Ajman, ARE; 4 Faculty of Dentistry, University of Khartoum, Khartoum, SDN; 5 Department of Oral and Maxillofacial Surgery, NHS England, Wolverhampton, GBR; 6 Department of General Dentistry, University of Khartoum, Khartoum, SDN

**Keywords:** anterior open bite, early orthodontic treatment, habit-breaking appliances, mixed dentition, molar intrusion, orthopedic intervention, overbite correction, systematic review

## Abstract

Anterior open bite (AOB) is a complex malocclusion prevalent in the mixed dentition, with multifactorial etiology involving skeletal, dental, and habitual components. Early intervention during this developmental stage is advocated to correct the malocclusion and guide growth, but the most effective and stable approaches remain debated. This systematic review aims to evaluate the efficacy of early orthodontic and orthopedic interventions for correcting AOB in the mixed dentition. A systematic search was conducted across four electronic databases (PubMed, Embase, Web of Science, and ClinicalTrials.gov) for randomized controlled trials (RCTs) published from January 2020 to October 2025. The review followed the Preferred Reporting Items for Systematic reviews and Meta-Analyses (PRISMA) guidelines. Eligible studies investigated children in the mixed dentition treated for AOB with any early intervention. Study selection, data extraction, and risk of bias assessment using the Cochrane RoB 2 tool were performed independently by two reviewers. A qualitative synthesis was undertaken due to clinical heterogeneity. Eight RCTs were included. All interventions, including bonded spurs, palatal cribs, the rapid molar intruder (RMI), and functional appliances, demonstrated statistically significant overbite improvement, with mean increases ranging from 2.8 to 4.9 mm. The primary mechanism of correction was dentoalveolar, involving incisor extrusion and lingual tipping. However, the RMI produced favorable skeletal changes, such as reduction of the mandibular plane angle, indicating true orthopedic mandibular rotation. One study reported high long-term stability (92.31%) for bonded spurs at a four-year follow-up. Another study found that interventions, particularly those with posterior build-ups, significantly improved oral health-related quality of life. The overall risk of bias was low for most included studies. Early intervention for AOB in the mixed dentition is highly effective. A range of appliances can successfully correct the overbite, primarily through dentoalveolar adaptation, though some can induce positive skeletal changes. Treatment also demonstrates promising long-term stability and improves patient-reported outcomes. The choice of appliance should be tailored to the specific etiology and desired treatment goals. These findings support a proactive interceptive approach in managing AOB.

## Introduction and background

Anterior open bite (AOB) is among the most challenging malocclusions to diagnose, manage, and maintain stability for in orthodontic practice [1]. It is defined as the absence of vertical overlap between the maxillary and mandibular incisors when the posterior teeth are in occlusion [2]. The etiology is multifactorial, involving skeletal, dental, functional, and environmental factors such as vertical growth anomalies, tongue-thrusting, prolonged digit or pacifier sucking, airway obstruction, and genetic predisposition [3]. Beyond aesthetic concerns, AOB can impair mastication, speech, and psychosocial well-being in affected children [2].

The mixed dentition phase represents a critical period for interceptive and orthopedic management [4]. Early intervention during this stage can modify adverse growth patterns, eliminate deleterious habits, and guide dentoalveolar development toward favorable outcomes [5]. Various orthopedic and orthodontic approaches, such as habit-breaking appliances, vertical-pull chin cups, high-pull headgear, rapid maxillary expansion (RME), bite blocks, and functional appliances, have been utilized to control vertical dimension and correct AOB [6]. However, the choice of intervention often depends on etiology, growth pattern, and patient compliance.

Despite ongoing clinical interest, the effectiveness and long-term stability of early interventions remain controversial [7]. Although several studies report short-term correction in overbite and facial aesthetics, relapse is frequent, particularly in patients with skeletal vertical dysplasia [8]. Previous systematic reviews on AOB are either outdated or lack a specific focus on the mixed dentition phase, resulting in limited evidence to guide clinicians on early management strategies.

Given these gaps, an updated and focused synthesis of available evidence is essential. Therefore, the present systematic review aims to critically evaluate and summarize current literature on early orthodontic and orthopedic interventions for AOB in the mixed dentition, emphasizing the effectiveness, stability, and limitations of treatment modalities to support evidence-based clinical decision-making.

## Review

Methodology

Protocol and Registration

This systematic review was conducted in accordance with the Preferred Reporting Items for Systematic reviews and Meta-Analyses (PRISMA) 2020 guidelines [9]. The review protocol was developed a priori to ensure transparency and reproducibility. Although the protocol was not formally registered in PROSPERO due to the limited availability of comparable prior evidence at the time of study conception, all methodological steps were conducted using a structured and predefined approach consistent with PRISMA standards [9]. This lack of protocol registration is acknowledged as a limitation of the present study.

Eligibility Criteria

Studies were selected based on predefined inclusion and exclusion criteria. Only randomized controlled trials (RCTs) were included in order to ensure a high level of evidence regarding the effectiveness of early orthodontic and orthopedic interventions for AOB correction in the mixed dentition. Eligible studies were those involving children in the mixed dentition phase who received early orthodontic or orthopedic treatment aimed at correcting AOB. Interventions could include habit-breaking appliances, functional or orthopedic devices, vertical-pull chin cups, bite blocks, RME, or other growth-modifying approaches. Studies focusing on adult patients, case reports, retrospective designs, reviews, or animal studies were excluded. Only articles published between January 2020 and October 2025 were considered, as this timeframe ensured that the review covered the most recent and clinically relevant literature reflecting current orthodontic practices and technologies. No language restrictions were applied.

Information Sources and Search Strategy

An extensive electronic literature search was performed across four major databases: PubMed (MEDLINE), Embase (Elsevier), Web of Science (Clarivate Analytics), and ClinicalTrials.gov. The search was conducted on October 1, 2025. A combination of Medical Subject Headings (MeSH) and free-text terms was used, including “anterior open bite”, “mixed dentition”, “early treatment”, “orthodontic”, “orthopedic”, “habit breaking”, and “randomized controlled trial”. Boolean operators (AND/OR) were applied to combine search terms appropriately. In addition, a manual citation search of the reference lists of all included studies and relevant reviews was performed to identify any additional eligible trials not captured in the electronic search.

Selection Process

All retrieved records were imported into EndNote X6 (Clarivate Analytics) for management and duplicate removal. Following deduplication, titles and abstracts were independently screened by two reviewers to identify potentially relevant studies. Full-text articles were then obtained and assessed for eligibility based on the inclusion and exclusion criteria. Any disagreements between reviewers during the selection process were resolved through discussion until consensus was reached.

Data Collection Process and Data Items

Data extraction was conducted independently by two reviewers using a standardized data extraction form. Extracted information included study characteristics (authors, year, and country), participant details (sample size, age range, and dentition stage), intervention type, comparator, treatment duration, outcome measures, and main findings. Primary outcomes included changes in overbite and vertical skeletal parameters, while secondary outcomes included relapse tendency, treatment stability, and patient compliance. Discrepancies in data extraction were resolved by consensus discussion.

Study Risk of Bias Assessment

The methodological quality of each included RCT was assessed using the Revised Cochrane Risk-of-Bias Tool for Randomized Trials (RoB 2) [10]. The tool evaluates bias across several domains, including the randomization process, deviations from intended interventions, missing outcome data, measurement of outcomes, and selection of the reported result. Each domain was judged as “low risk,” “some concerns,” or “high risk” of bias. Assessments were performed independently by two reviewers, and disagreements were resolved by discussion.

Synthesis Methods

Given the expected heterogeneity among studies in terms of intervention types, appliance designs, treatment durations, outcome measures, and follow-up periods, a quantitative meta-analysis was not conducted. Pooling of data was deemed inappropriate due to the lack of standardized outcome reporting and variations in measurement techniques and study designs. Instead, the findings were synthesized qualitatively, allowing for a detailed narrative comparison of treatment effectiveness and stability across studies.

Reporting Bias Assessment

Where applicable, reporting bias was evaluated by assessing the availability of study protocols, consistency between reported outcomes and registered trial outcomes, and selective outcome reporting. However, due to the small number of available RCTs and limited trial registrations, a formal statistical assessment of publication bias (e.g., funnel plot) was not feasible.

Results

Study Selection and Characteristics

**Figure 1:**
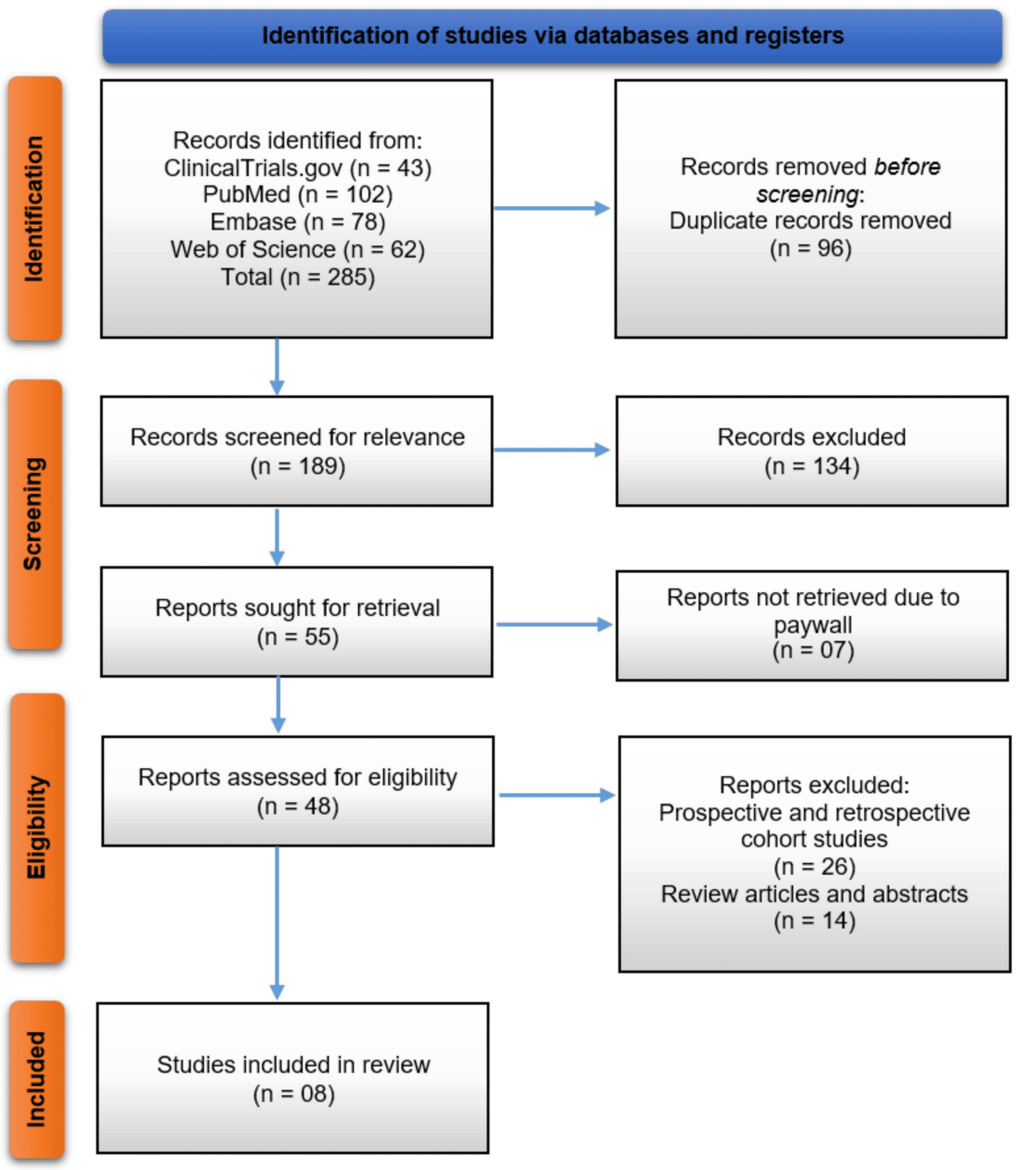
PRISMA flowchart of the study identification and selection process PRISMA, Preferred Reporting Items for Systematic reviews and Meta-Analyses

The systematic search across four electronic databases (ClinicalTrials.gov, PubMed, Embase, and Web of Science) initially identified 285 records. After the removal of 96 duplicate records, a total of 189 unique publications were screened by title and abstract for relevance. This screening process led to the exclusion of 134 records. The full texts of the remaining 55 articles were sought for retrieval; however, seven reports could not be accessed due to paywall restrictions. To minimize selection bias, we contacted the corresponding authors of these studies to request access, but no responses were received. Consequently, 48 full-text articles were assessed for eligibility. Of these, 26 were excluded for being prospective or retrospective cohort studies, and a further 14 were excluded as they were review articles or conference abstracts. Ultimately, eight RCTs met all inclusion criteria and were included in the final qualitative synthesis of this systematic review [11-18]. The study selection process is detailed in the PRISMA flow diagram (Figure 1).

The characteristics of these studies are summarized in Table 1. The included studies were published between 2021 and 2025. Geographically, the studies originated from Brazil [11,12,16,18], Syria [13,14], and Egypt [15,17]. All investigations focused on patients in the mixed dentition stage, with age ranges typically spanning from seven to 12 years. The sample sizes varied, with the smallest trial including 24 participants [12] and the largest comprising 75 participants distributed across three groups [17].

**Table 1:** Characteristics of included studies AOB, anterior open bite; ANB, A point-nasion-B point (cephalometric angle); BS, bonded spurs; CFPC, conventional fixed palatal crib; FHP, Frankfort horizontal plane; FMA, Frankfort-mandibular plane angle; L1/GoGn, long axis of lower incisor to Gonion-Gnathion plane; MSPC, miniscrew-supported palatal crib; OBB, open-bite bionator; OHRQOL, oral health-related quality of life; RPBP/C, removable posterior bite plane with tongue crib; RCT, randomized controlled trial; RMI, removable myofunctional inclined plane; S, spurs (bonded spurs only); SBU, spurs + posterior build-ups; SNA, Sella-Nasion-A point (cephalometric angle); SNB, Sella-Nasion-B point (cephalometric angle); TCR, two-arm, parallel-group clinical trial; U1/FHP, upper incisor to Frankfort horizontal plane; UCG, untreated control group

Author (year)	Country/region	Study design	Sample size (n)	Age range/dentition stage	Intervention type	Comparator/control	Treatment duration	Follow-up period	Primary outcome(s)
Aliaga-Del Castillo et al. (2022) [11]	Brazil	RCT	Experimental: 24; control: 25	7-11 years/mixed dentition	Bonded spurs + posterior build-ups	Bonded spurs alone	12 months	12 months	3D maxillary dentoalveolar changes (incisors and first molars)
Aliaga-Del Castillo et al. (2021) [12]	Brazil (university clinic, single-center)	TCR (two-arm, parallel-group)	24 (SBU), 25 (S)	7-11 years/mixed dentition	Bonded SBU	Bonded spurs alone (S)	12 months	12 months	Overbite change (mm)
Hasan et al. (2022) [13]	Syria	RCT (two-arm, parallel-group)	40	8-12 years/mixed dentition	RMI	UCG	9 months	9 months	Skeletal, dentoalveolar, and soft tissue changes
Mousa et al. (2021) [14]	Syria	RCT (two-arm, parallel-group)	40 (20 per group)	7.5-10.5 years/mixed dentition	OBB	RPBP/C	12 months	12 months	Skeletal and dentoalveolar variables; secondary: soft-tissue parameters on lateral cephalograms
Fouda et al. (2022) [15]	Egypt	RCT	26 (MSPC: 12, CFPC: 12)	8-11 years/mixed dentition	MSPC	CFPC	9 months	9 months	Amount of AOB closure, mesial movement of maxillary first molars
Aliaga-Del Castillo et al. (2024) [16]	Brazil	Randomized trial	49 (SBU: 24; S: 25)	7-11 years/mixed dentition	Lingual SBU	Lingual S	12 months	12 months	OHRQOL, functional adaptation, discomfort, and functional limitations
Alawy et al. (2025) [17]	Egypt	Randomized/interventional (three-group comparison)	75 (three groups: 25 each)	7-9 years/mixed dentition	Group I: prefabricated metal-bonded tongue tamers; Group II: customized composite-bonded spurs	Group III: conventional fixed palatal cribs	3 months	3 months	Overbite (model and cephalometric), overjet, cephalometric angles (SNA, SNB, ANB, FMA, U1/FHP, and L1/GoGn)
Utrago et al. (2025) [18]	Brazil	Prospective controlled study	BS group: 26; control group: 20	Mixed dentition, mean age BS: 8.06-13.3 years	Bonded spurs (BS)	Untreated subjects with AOB	1 year	4 years post-treatment	Overbite change, incisor extrusion, incisor tip changes, and relapse rate

The interventions evaluated were diverse, reflecting a range of early treatment approaches for AOB. These included appliances designed to modify tongue posture and function, such as bonded spurs (with or without posterior build-ups) [11,12,16,18], various types of palatal cribs (conventional fixed, miniscrew-supported, and prefabricated tongue tamers) [15,17], and a removable posterior bite plane with tongue crib (RPBP/C) [14]. Other studies investigated orthopedic appliances aimed at influencing vertical growth, such as the rapid molar intruder (RMI) [13] and the open-bite Bionator (OBB) [14]. Comparator groups typically consisted of either an alternative active intervention, a placebo, or an untreated control. Treatment duration ranged from three to 12 months, with follow-up periods generally aligning with the active treatment phase, though one study reported a long-term follow-up of four years post-treatment [18].

Primary Outcomes: Overbite Correction and Dentoalveolar Changes

The primary outcome across all studies was the change in overbite, measured in millimeters (mm). The statistical outcomes for this and other measures are detailed in Table 2. All active interventions demonstrated a statistically significant improvement in overbite compared to untreated control groups or baseline measurements.

**Table 2:** Summary of statistical outcomes of included studies AOB, anterior open bite; ANB, A point-Nasion-B point angle; CFPC, conventional fixed posterior crib; exp, experimental group; FHP, Frankfort horizontal plane; FMA, Frankfort mandibular plane angle; L1/GoGn, lower incisor to mandibular plane angle (Gonion-Gnathion plane); MD, mean difference; MSPC, modified simple posterior crib; NR, not reported; OBB, occlusal build-up; OHRQOL, oral health-related quality of life; RMI, removable molar intrusion appliance; RPBP/C, removable posterior bite plate/crib; S, spur; SBU, smart bite-up; SNA, Sella-Nasion-A point angle; SNB, Sella-Nasion-B point angle; SN:GoMe, Sella-Nasion to Gonion-Menton angle; Sum of Bjork, composite measure of vertical skeletal relationships; U1/FHP, upper incisor to Frankfort horizontal plane

Author (year)	Mean overbite change (mm)	Cephalometric change (°)	P-value/statistical significance	Effect size/CI	Key findings
Aliaga-Del Castillo et al. (2022) [11]	1.55-2.92 (exp) vs. 1.40-2.65 (comp)	First molars: -2.16° (lingual, exp) vs. buccal (comp)	<0.05	Molars: MD -0.13 mm (-0.38, 0.12); MD -0.31 mm (-0.51, -0.11); inclination MD -2.16° (-3.72, -0.60)	Similar incisor and molar displacement; build-ups caused medial and lingual molar changes
Aliaga-Del Castillo et al. (2021) [12]	~4 mm (both groups)	Maxilla: 1.31-1.55 mm; mandible: 1.31-1.55 mm; maxillary intermolar: -0.48 mm; mandibular intermolar: 0.26 mm	P < 0.05	Overbite MD -0.11 (-1.03 to 0.80); maxilla MD -0.24 (-0.91 to 0.44); mandible MD 0.29 (-0.39 to 0.96); max. intermolar -0.48 (-0.92 to -0.03); mand. intermolar 0.26 (0.004-0.52)	Both treatments improved AOB; SBU slightly decreased maxillary intermolar and increased mandibular intermolar, opposite in S. Plaque noted.
Hasan et al. (2022) [13]	RMI: 4.44 mm; control: 0.19 mm	SN:GoMe angle ↓ (RMI) vs. ↑ (Control) Sum of Bjork ↓ (RMI) vs. ↑ (Control)	p < 0.001	NR	RMI significantly increased overbite and intruded upper and lower first molars, leading to favorable skeletal, dentoalveolar, and soft tissue changes
Mousa et al. (2021) [14]	OBB: 4.91 ± 0.4; RPBP/C: 3.43 ± 0.3	Dentoalveolar extrusion and lingual tipping of incisors (statistically significant, P ≤ 0.05); no significant skeletal changes	Overbite difference: P = 0.003; dentoalveolar changes: P ≤ 0.05	NR	Both OBB and RPBP/C effective in correcting AOB; overbite closure mainly due to dentoalveolar changes; no significant difference in skeletal outcomes between the two appliances
Fouda et al. (2022) [15]	MSPC: 3.97 ± 1.44; CFPC: 3.97 ± 0.89	NR	Sig for molar movement	NR	Both appliances closed AOB similarly. CFPC showed more mesial movement of maxillary first molars (-1.42 ± 0.99 mm vs. -0.53 ± 0.32 mm MSPC)
Aliaga-Del Castillo et al. (2024) [16]	NR	NR	Func limits S: <0.001; discomfort: <0.001	OHRQOL: 0.69 (0.55-0.88); discomfort one week: 0.19 (0.13-0.28), 1 month: 0.02 (0.01-0.07)	SBU vs. S: easy adaptation; tongue discomfort ↓; func limits improved S; overall OHRQOL ↑
Alawy et al.(2025) [17	2.81-3.16 ↑	FMA -2.6, SNA -0.63 to -1.31, ANB -0.90 to -1.62, SNB 0.34-0.49, U1/FHP -1.76, L1/GoGn -2.54	Overjet reduction significant for tongue tamers vs. bonded spurs; other changes nonsignificant	NR	All interventions increased overbite; tongue tamers showed the highest overjet reduction and favorable skeletal changes; early treatment promoted correction and occlusal function
Utrago et al. (2025) [18]	During treatment: 4.53 ± 1.55 mm; four-year follow-up: 0.87 ± 1.17 mm	Upper incisors tip: 2.72 ± 1.25°; lower incisors tip: -2.24 ± 3.61°	Long-term overbite changes not significantly influenced by initial AOB severity or facial growth pattern (P = 0.831)	NR	Bonded spurs effective in treating AOB in mixed dentition, with high long-term stability (92.31%). Significant overbite increase and incisor extrusion observed compared to control

The magnitude of overbite change varied by appliance. Studies on the RMI and OBB reported the largest mean improvements, with 4.44 mm [13] and 4.91 mm [14], respectively. Bonded spurs, both alone and in combination with posterior build-ups, also produced substantial overbite increases, ranging from approximately 1.40 to 4.53 mm during active treatment [11,12,18]. Similarly, palatal cribs and tongue tamers showed effective overbite correction, with mean changes around 3.97 mm [15] and 2.81-3.16 mm [17].

The mechanisms of overbite correction were primarily dentoalveolar. Several studies reported significant extrusion and lingual tipping of the incisors [14,18]. Molar changes also played a critical role; for instance, the RMI achieved overbite closure primarily through the intrusion of upper and lower first molars [13]. In contrast, the study by Aliaga-Del Castillo et al. [11] found that the addition of posterior build-ups to bonded spurs resulted in medial and lingual movement of the maxillary first molars, a change not observed in the spurs-only group. Fouda et al. [15] noted that while both miniscrew-supported and conventional fixed palatal cribs closed the AOB effectively, the conventional crib was associated with significantly greater mesial movement of the maxillary first molars.

Skeletal and Cephalometric Changes

While the primary effects were dentoalveolar, some interventions induced favorable skeletal changes. The RMI, for example, demonstrated a significant reduction in the SN:GoMe angle and the sum of Bjork's angles, indicating a counter-clockwise rotation of the mandible and improvement in the skeletal vertical dimension [13]. Similarly, Alawy et al. [17] reported a reduction in the Frankfort-mandibular plane angle (FMA) and the ANB angle in groups treated with tongue tamers and bonded spurs, suggesting favorable skeletal modifications.

However, other studies found that skeletal contributions were minimal. Mousa et al. [14] concluded that the overbite correction achieved with both the OBB and the RPBP/C was mainly due to dentoalveolar changes, with no statistically significant skeletal differences between the two appliances. Utrago et al. [18] supported the notion of dentoalveolar dominance, finding that long-term stability was not significantly influenced by the initial skeletal pattern or facial growth type.

Patient-Centered Outcomes: Quality of Life and Stability

One study specifically investigated patient-centered outcomes [16]. Aliaga-Del Castillo et al. reported that treatment with bonded spurs, especially when combined with posterior build-ups (SBU), was associated with significant improvements in oral health-related quality of life (OHRQOL). Patients in the SBU group experienced less functional limitation and discomfort and adapted more easily compared to those treated with spurs alone (S).

Regarding stability, Utrago et al. [18] provided valuable long-term data. Their four-year follow-up study found that the significant overbite increase achieved with bonded spurs (4.53 ± 1.55 mm) was largely maintained, with a mean relapse of only 0.87 ± 1.17 mm, resulting in a high long-term stability rate of 92.31%. This suggests that early intervention with bonded spurs in the mixed dentition can produce durable results.

Risk of Bias Assessment

The methodological quality of the included studies was assessed using the Cochrane Risk of Bias (RoB 2) tool. The majority of the trials were judged to have a low overall risk of bias [11-14,16,18]. However, two studies raised some concerns [15,17]. Specifically, the trials by Fouda et al. [15] and Alawy et al. [17] were rated with some concerns regarding the overall risk of bias, primarily due to issues in the selection of the reported result, as their analysis plans were not preregistered in a publicly available protocol, creating the potential for selective outcome reporting. Furthermore, for these two studies [15,17], the randomization process also raised some concerns due to insufficient detail on the method of sequence generation and allocation concealment. Across all studies, the domains concerning deviations from intended interventions, missing outcome data, and measurement of the outcome were consistently judged as low risk (Table 3).

**Table 3:** Risk of Bias Assessment (Cochrane RoB 2 Tool)

Study (author and year)	D1: Randomization process	D2: Deviations from intended interventions	D3: Missing outcome data	D4: Measurement of the outcome	D5: Selection of the reported result	Overall risk of bias
Aliaga-Del Castillo et al. (2022) [11]	Low	Low	Low	Low	Low	Low
Aliaga-Del Castillo et al. (2021) [12]	Low	Low	Low	Low	Low	Low
Hasan et al. (2022) [13]	Low	Low	Low	Low	Low	Low
Mousa et al. (2021) [14]	Low	Low	Low	Low	Low	Low
Fouda et al. (2022) [15]	Some concerns	Low	Low	Low	Some concerns	Some concerns
Aliaga-Del Castillo et al. (2024) [16]	Low	Low	Low	Low	Low	Low
Alawy et al. (2025) [17]	Some concerns	Low	Low	Low	Some concerns	Some concerns
Utrago et al. (2025) [18]	Low	Low	Low	Low	Low	Low

Discussion

This systematic review comprehensively synthesized the evidence from eight RCTs evaluating the efficacy of early orthodontic and orthopedic interventions for the management of AOB in the mixed dentition. The findings collectively demonstrate that a range of therapeutic modalities, from simple behavioral appliances to complex orthopedic intruders, are effective in achieving significant overbite correction. The magnitude of this correction is substantial, with mean improvements ranging from approximately 2.8-4.9 mm, effectively closing the open bite in the growing patient [13-15,17]. A critical synthesis of the evidence reveals that the primary mechanism of action for most appliances is dentoalveolar, involving the extrusion and lingual tipping of the anterior teeth and strategic changes in molar position. However, certain interventions also demonstrate a capacity to elicit favorable skeletal changes, influencing the vertical dimension of the face. Furthermore, the review provides preliminary insights into patient-centered outcomes and long-term stability, offering a more holistic view of early AOB treatment than previously available.

The predominant dentoalveolar effect observed across most included studies underscores the plasticity of the alveolar process in children. The significant incisor extrusion and lingual inclination reported with bonded spurs and palatal cribs [14,18] are consistent with their proposed mechanism of disrupting aberrant tongue posture and facilitating the natural eruption of anterior teeth. This aligns with the historical work of Subtelny and Sakuda [19], who emphasized the role of dentoalveolar development in open bite etiology and correction. Similarly, the finding that posterior build-ups can induce medial and lingual movement of the maxillary molars [11] introduces a valuable 3D perspective, suggesting that these appliances do not merely act as passive tongue fences but actively influence the transverse and sagittal positions of the posterior teeth, potentially enhancing occlusal locking and stability. The comparison between miniscrew-supported and conventional palatal cribs by Fouda et al. [15] further refines our understanding; the similar overbite closure but differential molar movement highlights a trade-off between maximum anchorage control with skeletal support and the potential for spontaneous dentoalveolar adjustment with conventional designs. This finding resonates with studies on molar distalization, where miniscrews provide absolute anchorage, preventing the mesial movement often seen with conventional appliances.

Beyond dentoalveolar compensation, the results from studies employing the RMI [13] and, to a lesser extent, tongue tamers and bonded spurs [17], indicate that certain interventions can indeed modify the skeletal pattern. The reduction in the SN:GoMe angle and the sum of Bjork’s angles with the RMI [13] is a particularly noteworthy finding. This represents a true orthopedic effect, likely achieved through the intentional intrusion of maxillary molars, which induces a counterclockwise rotation of the mandible. This mechanism is a cornerstone of vertical control in orthodontics and is supported by previous research on temporary skeletal anchorage devices for molar intrusion in adolescent and adult patients. The findings of Hasan et al. [13] suggest that this powerful mechanism can be effectively harnessed even earlier, in the mixed dentition, to address the underlying skeletal component of AOB. Similarly, the reported reductions in FMA and ANB by Alawy et al. [17], while potentially involving a dentoalveolar component, point toward a favorable skeletal response. This contrasts with the conclusions of Mousa et al. [14], who found no significant skeletal changes with the OBB or the removable posterior bite plane with crib. This discrepancy may be attributed to the fundamental difference in the appliance mechanism; the RMI is a fixed, passive intruder applying continuous force, whereas functional appliances like the Bionator rely on patient compliance and neuromuscular adaptation, which may produce more variable outcomes. Our finding that skeletal change is achievable corroborates the work of Cozza et al. [20], who demonstrated similar mandibular counter-clockwise rotation with a modified quad-helix/crib combination, but extends it by showing that more predictable intrusion-driven effects are possible with modern skeletal anchorage.

A pivotal finding of this review is the long-term stability data provided by Utrago et al. [18]. The reported 92.31% stability rate four years post-treatment is exceptionally high and challenges the traditional skepticism surrounding the long-term efficacy of early AOB intervention. This remarkable stability can be attributed to the nature of the treatment effect. By using bonded spurs to correct the AOB primarily through incisor extrusion and by addressing the associated tongue habit, the therapy targets a key etiological factor. The resulting improvement in lip seal and oral posture likely creates a favorable environment for maintaining the corrected overbite. This finding is supported by a retrospective study by López-Gavito et al. [21], which identified the persistence of a non-adapted tongue posture as a primary factor for relapse. The high stability reported by Utrago et al. [18] suggests that early intervention, when it successfully modifies the soft tissue environment, can yield durable results, potentially reducing the need for more complex treatment in the permanent dentition.

Furthermore, the inclusion of patient-reported outcomes in this review marks a significant advancement in the appraisal of these interventions. The study by Aliaga-Del Castillo et al. [16] revealed that treatment, particularly with posterior build-ups, was associated with significant improvements in OHRQOL, reduced discomfort, and fewer functional limitations over time. This is a critical consideration for clinical practice. While the objective cephalometric and model analyses confirm clinical efficacy, the patient's subjective experience ultimately influences adherence and overall treatment success. The finding that children adapt well to these fixed appliances and report improved quality of life dispels a common concern among clinicians regarding patient tolerance. This aligns with a growing body of evidence in orthodontics, such as that from Liu et al. [22], which emphasizes the importance of evaluating OHRQOL alongside traditional biomechanical outcomes.

When interpreting these promising results, the methodological quality of the underlying evidence must be considered. The majority of the included trials were judged to have a low risk of bias, which strengthens the validity of our conclusions [11-14,16,18]. However, the presence of “some concerns” in two studies [15,17], primarily due to a lack of preregistered protocols, introduces a degree of caution regarding the potential for selective outcome reporting. Nonetheless, the consistency of the primary outcome, overbite improvement, across all studies, regardless of their risk of bias, reinforces the central finding that these interventions are effective.

Clinical Implications

For clinicians, this review highlights that early orthodontic and orthopedic interventions can effectively manage AOB in the mixed dentition. Bonded spurs and palatal cribs demonstrate high long-term stability, particularly when tongue habits are addressed, while posterior build-ups improve overbite through dentoalveolar and transverse adjustments. Skeletal anchorage-based appliances such as the RMI offer measurable vertical skeletal improvements, including mandibular counter-clockwise rotation and molar intrusion, which may be advantageous in patients with predominant skeletal open bite. Functional appliances like the Bionator and removable posterior bite planes can be effective but may yield more variable skeletal responses due to reliance on patient compliance. Additionally, treatment positively influences patient-reported outcomes, improving OHRQOL, comfort, and functional adaptation, which supports adherence and overall treatment success.

Limitations

This systematic review has several limitations. First, only eight RCTs were included, and a meta-analysis was not feasible due to clinical and methodological heterogeneity, limiting quantitative comparison between interventions. Second, most studies were conducted in Brazil and the Middle East, potentially introducing population bias and limiting generalizability. Third, the protocol was not registered in PROSPERO, which may increase the risk of selective reporting. Fourth, language and publication bias cannot be excluded, as only English-language and published studies were considered. Finally, the interaction between different growth patterns and treatment response was not extensively explored in the included studies, and comparisons with previous systematic reviews (prior to 2020) remain limited, representing important areas for future research.

## Conclusions

Early intervention for AOB in the mixed dentition is effective, primarily through dentoalveolar changes but with certain appliances capable of inducing favorable skeletal modifications. Appliances such as bonded spurs, palatal cribs, and the RMI all produce significant overbite correction, with high long-term stability demonstrated for bonded spurs. The choice of appliance should be guided by the specific etiology of the AOB, the desired treatment effects (dentoalveolar versus skeletal), and patient-specific factors, including tolerance and quality of life impacts. The findings advocate for a proactive approach to AOB management in the mixed dentition, as these interventions can effectively correct the malocclusion, improve oral function and quality of life, and potentially mitigate the need for more invasive treatments later in life. While these results are promising, larger multicenter RCTs with standardized reporting are needed to validate these findings. Future research should prioritize long-term, multicenter randomized trials with standardized outcome sets to allow for direct comparisons and a more precise understanding of the factors predicting treatment success and stability.
